# Prediction of a positive circumferential resection margin at surgery following neoadjuvant chemotherapy for adenocarcinoma of the oesophagus

**DOI:** 10.1002/bjs5.50211

**Published:** 2019-08-22

**Authors:** W. R. C. Knight, C. Yip, W. Wulaningsih, A. Jacques, N. Griffin, J. Zylstra, M. Van Hemelrijck, N. Maisey, A. Gaya, C. R. Baker, M. Kelly, J. A. Gossage, J. Lagergren, D. Landau, V. Goh, A. R. Davies, on behalf of the Guy's and St Thomas' Oesophago‐Gastric Research Group, S. Ngan, A. Qureshi, H. Deere, M. Green, F. Chang, U. Mahadeva, B. Gill‐Barman, S. George, J. Dunn, S. Zeki, J. Meenan, O. Hynes, G. Tham, C. Iezzi

**Affiliations:** ^1^ Department of Surgery, Guy's and St Thomas' Oesophago‐Gastric Centre, King's College London; ^2^ School of Cancer and Pharmaceutical Sciences, King's College London; ^3^ School of Biomedical Engineering and Imaging Sciences, King's College London; ^4^ Cancer Epidemiology and Population Health Associated Research Group, King's College London; ^5^ Department of Radiology, Guy's and St Thomas' Hospital, London, UK; ^6^ Department of Oncology, Guy's and St Thomas' Hospital, London, UK; ^7^ Upper Gastrointestinal Surgery, Department of Molecular Medicine and Surgery, Karolinska Institutet, Stockholm, Sweden

## Abstract

**Background:**

A positive circumferential resection margin (CRM) has been associated with higher rates of locoregional recurrence and worse survival in oesophageal cancer. The aim of this study was to establish if clinicopathological and radiological variables might predict CRM positivity in patients who received neoadjuvant chemotherapy before surgery for oesophageal adenocarcinoma.

**Methods:**

Multivariable analysis of clinicopathological and CT imaging characteristics considered potentially predictive of CRM was performed at initial staging and following neoadjuvant chemotherapy. Prediction models were constructed. The area under the curve (AUC) with 95% confidence intervals (c.i.) from 1000 bootstrapping was assessed.

**Results:**

A total of 223 patients were included in the study**.** Poor differentiation (odds ratio (OR) 2·84, 95 per cent c.i. 1·39 to 6·01) and advanced clinical tumour status (T3–4) (OR 2·93, 1·03 to 9·48) were independently associated with an increased CRM risk at diagnosis. CT‐assessed lack of response (stable or progressive disease) following chemotherapy independently corresponded with an increased risk of CRM positivity (OR 3·38, 1·43 to 8·50). Additional CT evidence of local invasion and higher CT tumour volume (14 cm^3^) improved the performance of a prediction model, including all the above parameters, with an AUC (c‐index) of 0·76 (0·67 to 0·83). Variables associated with significantly higher rates of locoregional recurrence were pN status (*P* = 0·020), lymphovascular invasion (*P* = 0·007) and poor response to chemotherapy (Mandard score 4–5) (*P* = 0·006). CRM positivity was associated with a higher locoregional recurrence rate, but this was not statistically significant (*P* = 0·092).

**Conclusion:**

The presence of advanced cT status, poor tumour differentiation, and CT‐assessed lack of response to chemotherapy, higher tumour volume and local invasion can be used to identify patients at risk of a positive CRM following neoadjuvant chemotherapy.

## Graphical abstract

A positive circumferential resection margin (CRM) has been shown to be an adverse prognostic indicator after oesophagectomy for adenocarcinoma. Clinical tumour status, tumour volume, poor differentiation, lack of response following chemotherapy and radiological assessment of invasion can be used to predict CRM positivity in oesophageal adenocarcinoma.

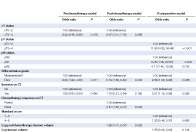

CRM assessment is still important

## Abstract (es)

**Antecedentes:**

Un margen de resección circunferencial (*circumferential resection margin*, CRM) positivo se ha asociado con tasas más elevadas de recidiva locorregional y peor supervivencia en el cáncer de esófago. El objetivo de este estudio fue establecer si las variables clínico‐patológicas y radiológicas podrían predecir la positividad del CRM en el adenocarcinoma de esófago tras quimioterapia neoadyuvante antes de la cirugía.

**Métodos:**

Se realizó un análisis multivariable de las características clínico‐patológicas y de la tomografía computarizada (*computed tomography*, CT) que se consideraron potencialmente predictivas de CRM en la estadificación inicial y tras la quimioterapia neoadyuvante. Se construyeron modelos de predicción. Se evaluó el área bajo la curva (*area under curve*, AUC) con el i.c. del 95% a partir de 1.000 muestras *bootstrap*.

**Resultados:**

Se incluyeron 223 pacientes en el estudio. Una pobre diferenciación (razón de oportunidades, *odds ratio*, OR 2,84, i.c. del 95% 1,39‐6,01) y un estadio clínico T avanzado (T3‐4) (OR 2,93, i.c. del 95% 1,03‐9,48) se asociaron de forma independiente con un riesgo aumentado de CRM en el diagnóstico. La falta de respuesta en la CT (estable o enfermedad en progresión) tras la quimioterapia se correspondía de forma independiente con un riesgo aumentado de CRM positivo (OR 3,38, i.c. del 95% 1,43‐8,50). Además, la evidencia por CT de invasión local y un mayor volumen del tumor en CT (14 cm^3^) mejoraron el funcionamiento del modelo predictivo, incluyendo todos los parámetros previamente señalados; con AUC (índice c) de 0,76 (0,68‐0,83). Las variables asociadas de forma significativa con tasas más elevadas de recidiva locorregional fueron el estado de los ganglios linfáticos patológicos (*P* = 0,002), la invasión linfovascular (*P* = 0,007) y la respuesta pobre a la quimioterapia (Mandard 4 y 5 (*P* = 0,006)). La positividad del CRM se asoció con una tasa de recidiva locorregional más elevada pero sin alcanzar significación estadística (*P* = 0,09).

**Conclusión:**

La presencia de un estadio clínico T avanzado, tumor pobremente diferenciado, falta de respuesta a la quimioterapia en la TC, mayor volumen del tumor en la TC e invasión local pueden ser utilizados para identificar pacientes en riesgo de un CRM positivo tras quimioterapia neoadyuvante.

## Introduction

The 5‐year overall survival (OS) rate for patients undergoing oesophagectomy is usually in the range of 17–40 per cent^1^, ^2^. In patients with oesophageal adenocarcinoma who are thought to have only locoregional disease, neoadjuvant therapy is frequently recommended^3^, ^4^. The survival advantage seen in the OEO‐2^5^ and MAGIC^6^ trials led to the widespread use of neoadjuvant chemotherapy. The CROSS trial^7^ demonstrated a survival advantage following neoadjuvant chemoradiotherapy for patients with squamous cell carcinoma, but this benefit was less evident in patients with adenocarcinoma.

A positive circumferential resection margin (CRM) on histopathological analysis is associated with poorer long‐term survival in patients who have undergone resection for oesophageal cancer^8^, ^9^, ^10^. The mechanism by which a positive CRM impacts survival is complex. Some studies^9^, ^11^, ^12^ have shown CRM positivity to be associated with increased rates of locoregional recurrence, whereas others have shown no independently increased risk^13^, ^14^. The relationship between a positive CRM and poorer survival is more pronounced in patients with fewer lymph node metastases (better prognosis groups) and also in those undergoing surgery alone^8^, ^15^, ^16^. In patients receiving either neoadjuvant chemotherapy^4^, ^17^ or chemoradiotherapy^7^, ^18^ the rates of CRM positivity are reduced, although CRM as an independent prognostic marker appears to be of less importance, presumably because of the additional systemic benefits afforded by multimodality treatment^16^.

Preoperative CRM prediction has proved an effective strategy in tailoring neoadjuvant and surgical strategies in rectal cancer, reducing rates of margin positivity and locoregional recurrence^19^, ^20^. This approach has not yet been explored in oesophageal adenocarcinoma. Oesophageal CRM prediction may be useful in stratifying patients for further therapy, with the aim of improving local response in high‐risk patients while avoiding potentially toxic therapy in low‐risk patients. The aim of this study was to establish whether preoperative clinicopathological and CT‐based radiological variables might predict a positive CRM in patients with oesophageal adenocarcinoma undergoing neoadjuvant chemotherapy before surgery.

## Methods

Consecutive patients who underwent potentially curative oesophagectomy from 2000 to 2012 were identified from an institutional database, with electronic CT images available for review. An initial analysis was performed to identify CT‐based radiological parameters that predicted CRM positivity in patients undergoing oesophagectomy between 2000 and 2007. This analysis was performed to isolate CT‐based radiological variables that might be useful in the main analysis. These CT‐based parameters were then combined with radiological metrics available only in the later study period (tumour volume, response to chemotherapy).

The main study cohort consisted of patients with adenocarcinoma only. These patients had all received neoadjuvant chemotherapy before oesophagectomy between 2007 and 2012. Patients were followed up in surgical and/or oncological clinics, with relevant information, including survival and recurrence, recorded in a central database. Hospital, cancer registry and general practitioner records also contributed to survival data. Outcomes of all patients were updated in February 2016.

Locoregional recurrence was defined as any disease (luminal or nodal) identified on endoscopy or imaging within the surgical resection field. The primary outcome of the study was a positive CRM as defined by the Royal College of Pathologists guidelines^21^ of tumour within 1 mm of the cut margin. The secondary outcome was the presence of locoregional recurrence either in isolation or as part of a mixed recurrence pattern.

Staging investigations included oesophagogastroduodenoscopy with biopsy, CT with intravenous contrast, [^18^F]fluorodeoxyglucose PET–CT and endoscopic ultrasonography (EUS). Routine fine‐needle aspiration of lymph nodes was not carried out during EUS. Patients with junctional tumours or those with evidence of disease below the diaphragm (primary tumour or lymph nodes) also underwent staging laparoscopy. Final clinical status was agreed by multidisciplinary consensus based on review of all staging investigations, with tumour status normally determined by EUS in the event of a discrepancy with CT findings.

Neoadjuvant chemotherapy consisted of standard platinum‐ and fluoropyrimidine‐based regimens as supported by RCT evidence^5^. Patients judged to have T2 status or above, or lymph node positivity, were considered for neoadjuvant chemotherapy. Patients had a further CT scan after neoadjuvant chemotherapy to assess response and confirm operability.

Transthoracic oesophagectomy (TTO) was performed by the Ivor Lewis or left thoracoabdominal approach with two‐field lymphadenectomy. Transhiatal oesophagectomy (THO) was performed in patients with lower oesophageal tumours, in whom dissection of the primary could be achieved under direct vision from the abdomen, along with an abdominal and lower mediastinal lymphadenectomy. The fat pad between the pericardium and the oesophagus was excised together with strips of right and left mediastinal pleura in continuity with the oesophagus.

For the initial radiological analysis, CT parameters were assessed retrospectively by an experienced gastrointestinal radiologist blinded to the outcome. In cases of pleural contact, pleural thickening adjacent to the tumour was recorded. Univariable and multivariable analyses of the association between radiological parameters and the risk of positive CRM involvement were done. Evidence of invasion, pleural thickening and aortic contact of more than 90° were combined as a single variable (invasion on CT).

In the main analysis cohort, longest axial diameter (LAD) and CT‐assessed invasion, contact and tumour volume were evaluated in prechemotherapy scans. These parameters were reassessed after chemotherapy along with radiological response to chemotherapy. Response was categorized as: response on CT, when there was downstaging or decrease in size of the primary tumour, or decrease in the size or number of involved nodes; or lack of response on CT, when there was no change in appearance of the primary tumour or involved nodes, or when there was evidence of progressive disease. For the purpose of modelling, postchemotherapy tumour volume was chosen over LAD as a representation of tumour dimension.

CRM predictors were analysed at three time points. The first two analyses (before neoadjuvant chemotherapy, after neoadjuvant chemotherapy) used clinical and radiological variables that would have been available at the time to construct clinically useful prediction models. The third analysis (postsurgical) used additional pathological variables available after surgery to establish the strongest standard CRM predictors overall. The purpose of this final analysis was to determine how the inaccuracies of staging modalities, compared with pathological results, had an impact on the accuracy of the models.

Variables used in the preneoadjuvant analysis were: cT and cN status (as determined by the multidisciplinary team from CT and EUS at diagnosis), grade (tumour differentiation), prechemotherapy radiological variables (LAD, tumour volume and invasion on CT). Variables used in the postneoadjuvant analysis were: intermediate ycT and cN status (from postchemotherapy CT), grade (tumour differentiation) and postchemotherapy radiological variables (LAD, tumour volume and CT response to chemotherapy). Additional variables used in the postsurgical analysis were: THO or TTO, ypT and ypN status, grade (tumour differentiation), lymphovascular invasion (yes or no) and response to chemotherapy (Mandard score).

### Statistical analysis

Continuous variables with skewed distributions were log‐transformed. Logistical regression was conducted to calculate odds ratios (ORs) and their 95 per cent c.i. of margin positivity by potential predictors. Univariable analyses for each predictor were conducted, and associations were deemed significant at *P* < 0·050. Multivariable modelling adjusting for potential confounders was conducted at the same time points. Statistical analysis was performed using R version 3.3.2 (R Foundation for Statistical Computing, Vienna, Austria).

A receiver operating characteristic (ROC) curve was used to present the final model performance. The concordance index (c‐index) and its 95 per cent c.i., obtained from cross‐validation with 1000 random resamples, was obtained to assess model discrimination. The c‐index reflects the proportion of pairs of patients (with opposing outcomes), where the patient who actually experiences the adverse outcome has a higher probability of the given outcome using the prediction model. The c‐index should have a 95 per cent c.i. that does not include 0·5. Using optimal cut‐off points identified from the ROC curve, sensitivity, specificity, and positive and negative predictive values were calculated. A final model was constructed with five postchemotherapy variables showing stepwise improvement of the area under the curve (AUC) with each variable. This included ORs (with 95 per cent c.i.) for CRM positivity with each additional parameter regardless of order. Odds of CRM positivity were also calculated with a formula derived from the regression model. For analysis locoregional recurrence rates, chi‐squared test was used.

## Results

A total of 223 patients were included in the analysis, 68 of whom were part of an initial radiological parameter cohort that included patients with adenocarcinoma, adenosquamous carcinoma and squamous cell carcinoma receiving neoadjuvant chemotherapy and surgery, or surgery alone. The univariable analysis of all parameters assessed in the initial radiological analysis of these 68 patients is summarized in *Table* *S1* (supporting information). These were assessed independently of tumour status. Invasion of adjacent structures (*P* = 0·030), contact with adjacent structures (*P* = 0·050), circumferential aortic contact greater than 90° (*P* = 0·050), pleural thickening (*P* = 0·030) and longest transaxial tumour dimension (*P* = 0·003) were associated with an increased risk of CRM positivity. The radiologist's prediction of CRM status was also associated with an increased risk of a positive CRM (*P* = 0·030). The presence of enlarged lymph nodes was not statistically significant (*P* = 0·070). When invasion, aortic contact and pleural thickening were included as a single variable (invasion on CT), this predicted CRM positivity in the multivariable analysis (OR 4·0, 95 per cent c.i. 1·23 to 16·08; *P* = 0.003). The AUC for these three variables for CRM positivity was 0·76 (0·64 to 0·88). The parameter of invasion on CT was thus brought forward to the main analysis.

The main analysis cohort consisted of 155 patients with adenocarcinoma who underwent neoadjuvant chemotherapy. Patient demographics, rates of CRM positivity and rates of locoregional recurrence according to each variable are outlined in *Table* 1. The majority of patients were men (82·6 per cent), and the mean age was 63·1 years. Most patients (81·9 per cent) were staged as cT3–4 before commencement of neoadjuvant chemotherapy.

**Table 1:** Patient demographics, rates of circumferential resection margin positivity and rates of locoregional recurrence

	Total (*n* = 155)	Positive CRM (*n* = 65)	Negative CRM (*n* = 90)	*P* *	Local recurrence (*n* = 35)	*P* *
**Mean age at operation (years)**	63·1					
**Sex**				0·250		0·038
F	27	14 (52)	13 (48)		2 (7)	
M	128	51 (39·8)	77 (60·2)		33 (25·8)	
**Oesophagectomy approach**				0·035		0·259
Transhiatal	75	25 (33)	50 (67)		14 (19)	
Transthoracic	80	40 (50)	40 (50)		21 (26)	
**cT status**				0·012		0·737
cT1–2	28	7 (25)	21 (75)		6 (21)	
cT3–4	127	65 (51·2)	62 (48·8)		29 (22·8)	
**cN status**				0·902		0·706
cN negative	30	12 (40)	18 (60)		6 (20)	
cN positive	125	53 (42·4)	72 (57·6)		29 (23·2)	
**pT status**				< 0·001		0·279
pT0–2	65	8 (12)	57 (88)		12 (19)	
pT3–4	90	57 (63)	33 (37)		23 (26)	
**pN status**				< 0·001		0·020
pN0	61	11 (18)	50 (82)		7 (11)	
pN1	37	20 (54)	17 (46)		10 (27)	
pN2–3	57	34 (60)	23 (40)		18 (32)	
**Tumour grade**				0·008		0·799
Moderately/well differentiated	79	25 (32)	54 (68)		16 (20)	
Poorly differentiated	76	40 (53)	36 (47)		19 (25)	
**Lymphovascular invasion**				< 0·001		0·007
No	66	14 (21)	52 (79)		8 (12)	
Yes	89	50 (56)	39 (44)		27 (30)	
**Mandard score**				0·019		0·006
1–3	52	15 (29)	37 (71)		5 (10)	
4–5	103	50 (48·6)	53 (51·5)		30 (29·1)	
**Longest transaxial dimension (cm)**				0·217		0·954
≥ 2·6	65	31 (47)	34 (52)		13 (20)	
< 2·6	90	34 (37)	56 (62)		22 (24)	
**Postchemotherapy tumour volume (cm** ^**3**^ **)**				0·0002		0·594
≥ 14	78	44 (56)	34 (44)		19 (24)	
< 14	77	21 (27)	56 (73)		16 (21)	
**CT estimation of chemotherapy response**				0·0094		0·660
Partial	49	13 (27)	36 (74)		10 (20)	
None	106	52 (48·6)	54 (51·4)		25 (23·6)	
**CT evidence of invasion**				0·110		0·067
Yes	51	26 (51)	25 (49)		16 (31)	
No	104	39 (37·5)	65 (62·5)		19 (18·3)	
**CRM positivity**						0·092
Yes	65				19 (29)	
No	90				16 (18)	

Values in parentheses are percentages. CRM, circumferential resection margin.

* χ^2^ test.

Univariable and multivariable analyses of predictors available at the time of diagnosis before chemotherapy are shown in *Table* 2. Poor differentiation (OR 2·84, 95 per cent c.i. 1·39 to 6·01; *P* = 0·005) and cT3–4 status (OR 2·93, 1·03 to 9·48; *P* = 0·05) independently increased the risk of a positive CRM in multivariable analysis. Evidence of invasion on CT before neoadjuvant chemotherapy did not increase the risk (OR 1·42, 0·63 to 3·23; *P* = 0·493).

**Table 2:** Univariable and multivariable analysis of circumferential resection margin positivity adjusting for variables available at the three time points

	**Before chemotherapy**				**After chemotherapy**				**After surgery (pathological)**			
	**Univariable**		**Multivariable**		**Univariable**		**Multivariable**		**Univariable**		**Multivariable**	
	**OR**	***P***	**OR**	***P***	**OR**	***P***	**OR**	***P***	**OR**	***P***	**OR**	***P***
**Age at operation**	0·95 (0·98, 1·00)	0·112	0·98 (0·95, 1·02)	0·346	0·95 (0·98, 1·00)	0·112	0·99 (0·96, 1·03)	0·716	0·95 (0·98, 1·00)	0·11	0·99 (0·94, 1·03)	0·57
**Sex**												
F	1·00 (reference)		1·00 (reference)		1·00 (reference)		1·00 (reference)		1·00 (reference)		1·00 (reference)	
M	0·70 (0·33, 1·50)	0·355	0·72 (0·28, 1·87)	0·500	0·70 (0·33, 1·50)	0·355	0·39 (0·14, 1·08)	0·073	0·70 (0·33, 1·50)	0·36	0·28 (0·07, 1·00)	0·06
**Surgical approach**												
TTO									1·00 (reference)		1·00 (reference)	
THO									0·50 (0·28, 0·86)	0·01	0·78 (0·31, 1·98)	0·60
**Tumour status**												
T1/2	1·00 (reference)		1·00 (reference)		1·00 (reference)		1·00 (reference)		1·00 (reference)		1·00 (reference)	
T3–4	1·24 (1·04, 1·48)	0·022	2·93 (1·03, 9·48)	0·054	1·12 (0·61, 2·08)	0·760	1·18 (0·57, 2·48)	0·657	16·36 (7·83, 37·88	< 0·001	14·04 (4·61, 52·14)	< 0·001
**cN status**												
Positive	1·00 (reference)		1·00 (reference)		1·00 (reference)		1·00 (reference)					
Negative	1·06 (0·90, 1·25)	0·469	0·82 (0·33, 2·05)	0·660	1·28 (0·66, 2·56	0·469	1·03 (0·41, 2·62)	0·956				
**pN category**												
pN0									1·00 (reference)		1·00 (reference)	
pN1									4·79 (2·25, 10·54)	< 0·001	5·32 (1·54, 20·41)	
pN2–3									7·89 (3·92, 16·59)	< 0·001	3·59 (1·10, 12·46)	0·011
**Preoperative differentiation**												
Moderate	1·00 (reference)		1·00 (reference)		1·00 (reference)		1·00 (reference)		1·00 (reference)		1·00 (reference)	
Poor	1·65 (0·94, 2·90)	0·082	2·84 (1·39, 6·01)	0·005	1·65 (0·94, 2·90)	0·082	3·58 (1·67, 8·04)	0·001	2·16 (1·25, 3·77)	0·006	2·96 (1·14, 8·21)	0·030
**Chemotherapy response on CT**												
Partial					1·00 (reference)		1·00 (reference)					
None					2·62 (1·32, ‐5·40)	0·007	3·38 (1·43, 8·50)	0·007				
**Mandard score**												
1–3									1·00 (reference)		1·00 (reference)	
4–5									3·88 (2·07, 7·57)	< 0·001	0·47 (0·14, 1·42)	0·360
**Log longest transaxial diameter**	1·32 (0·93, 1·91)	0·132	0·99 (0·60, 1·62)	0·978	2·03 (1·38, 3·14)	< 0·001	1·32 (0·67, 2·70)	0·425	2·03 (1·38, 3·14)	< 0·001	2·20 (0·49, 2·36)	0·087
**Log tumour volume**	0·99 (0·94, 1·05)	0·765	0·83 (0·60, 1·11)	0·220	1·87 (1·22, 3·03)	0·007	1·42 (0·86, 2·56)	0·206	1·87 (1·22, 3·03)	0·007	1·05 (0·49, 2·36)	0·889
**Lymphovascular invasion**												
No									1·00 (reference)		1·00 (reference)	
Yes									4·22 (2·36, 7·70)	< 0·001	3·75 (1·28, 11·81)	0·019
**Invasion on CT**												
No	1·00 (reference)		1·00 (reference)		1·00 (reference)		1·00 (reference)		1·00 (reference)		1·00 (reference)	
Yes	1·25 (0·66, 2·33)	0·493	1·42 (0·63, 3·23)	0·394	2·22 (1·23, 4·05)	0·008	1·50 (0·62, 3·73)	0·372	2·22 (1·23, 4·05)	0·008	3·06 (0·93, 5·80)	0·057

Values in parentheses are 95 per cent confidence intervals. OR, odds ratio; TTO, transthoracic; THO, transhiatal.

Univariable and multivariable analyses of predictors available following chemotherapy are shown in *Table* 2. All radiological parameters were significant in the univariable analysis (invasion on CT, *P* = 0·008; postchemotherapy tumour volume, *P* = 0·007; LAD, *P* < 0·001). Poor differentiation (OR 3·58, 1·67 to 8·04; *P* = 0·001) and no evidence of response to chemotherapy on CT (OR 3·38, 1·43 to 8·50; *P* = 0·007) independently predicted a positive CRM in multivariable analysis. Postchemotherapy ycT status was not statistically significant in univariable analysis (ycT3–4, *P* = 0·760).

Univariable and multivariable analyses of pathological variables available after surgery are shown in *Table* 2. Independent predictors of CRM positivity included pT3–4 disease (*P* < 0·001), pN1 disease (*P* = 0·011), pN2–3 disease (*P* = 0·037), poor differentiation (*P* = 0·030) and lymphovascular invasion (*P* = 0·019).

A summary of all the prediction models is shown in *Table* 3, and sensitivities, specificities, cut‐off values and positive and negative predictive values are given in *Table* 4. The prechemotherapy prediction model was constructed using three variables available at the time of diagnosis: cT status, tumour grade and evidence of invasion on CT. This had an AUC (c‐index) of 0·64 (95 per cent c.i. 0·56 to 0·72).

**Table 3:** Circumferential resection margin prediction models in oesophageal adenocarcinoma adjusted for variables available at the three time points

	Prechemotherapy model		Postchemotherapy model		Postoperative model	
	Odds ratio	*P*	Odds ratio	*P*	Odds ratio	*P*
**cT status**						
cT0–2	1·00 (reference)		1·00 (reference)			
cT3–4	2·32 (0·95, 6·29)	0·078	2·67 (1·01, 7·70)	0·055		
**pT status**						
pT0–2					1·00 (reference)	
pT3–4					11·58 (4·23, 36·46)	< 0·001
**pN status**						
pN0					1·00 (reference)	
pN1					6·05 (1·96, 20·57)	0·002
pN2					4·11 (1·40, 12·85)	0·012
**Differentiation grade**						
Moderate/well	1·00 (reference)		1·00 (reference)		1·00 (reference)	
Poor	2·30 (1·22, 4·39)	0·011	3·16 (1·52, 6·80)	0·003	2·56 (1·09, 6·29)	0·035
**Invasion on CT**						
No	1·00 (reference)		1·00 (reference)		1·00 (reference)	
Yes	1·22 (0·64, 2·34)	0·540	1·78 (0·81, 3·94)	0·152	3·01 (1·18, 8·26)	0·025
**Chemotherapy response on CT**						
Partial			1·00 (reference)			
None			3·47 (1·58, 8·07)	0·003		
**Mandard score**						
1–3					1·00 (reference)	
4–5					1·22 (0·46, 3·37)	0·695
**Log postchemotherapy tumour volume**			1·56 (1·01, 2·57)	0·062		
**Log tumour volume**					1·59 (0·88, 3·00)	0·144

Values in parentheses are 95 per cent confidence intervals.

**Table 4:** Sensitivity, specificity, cut‐off and predictive values for the three models

	Prechemotherapy model	Postchemotherapy model	Postoperative model
AUC	0·64 (0·56, 0·72)	0·75 (0·67, 0·82)	0·86 (0·80, 0·91)
Sensitivity (%)	55·1	76·9	81·0
Specificity (%)	67·7	64·4	80·0
Negative predictive value (%)	55·1	60·7	73·1
Positive predictive value (%)	67·7	68·7	83·7

Values in parentheses are 95 per cent confidence intervals. AUC, area under the receiver operating characteristic curve.

The postchemotherapy prediction model was constructed using five variables available in the preoperative, postchemotherapy period: cT status, tumour grade, invasion and chemotherapy response on CT, and postchemotherapy tumour volume. The AUC (c‐index) was 0·75 (95 per cent c.i. 0·67 to 0·82). For comparison, a six‐parameter prediction model was constructed using additional pathological variables only available after surgery (*Table* 3). The AUC (c‐index) was 0·86 (0·80 to 0·91).

*Table* 5 shows the postchemotherapy model with tumour volume dichotomized at 14 cm^3^ (median value). Each variable is shown with a stepwise improvement of AUC. With a tumour volume greater than 14 cm^3^, the model reached a cumulative AUC of 0·76 (0·68 to 0·83). The hazard ratio of CRM positivity was 2·50 (1·72 to 3·77) for each additional variable included, regardless of order. The probability of CRM positivity was calculated from the formula derived from the regression model:

**Table 5:** Circumferential resection margin positivity model showing postchemotherapy variables and incremental area under the curve improvement

	Variable	Cumulative AUC
Initial clinical tumour status	cT3 category or above	0·56 (0·51, 0·62)
Tumour behaviour	Poor differentiation	0·63 (0·55, 0·71)
Tumour response to chemotherapy	No response on CT	0·71 (0·63, 0·79)
Postchemotherapy tumour volume	Tumour volume > 14 cm^3^	0·75 (0·67, 0·82)
Radiological assessment	Invasion on CT (invasion or pleural thickening)	0·76 (0·67, 0·83)

Values in parentheses are 95 per cent confidence intervals. Hazard ratio of circumferential resection margin positivity: 2·50 (95 per cent c.i. 1·72, 3·77) for each additional point, regardless of order. AUC, area under the receiver operating characteristic curve.

exp(−3·12 (effect at baseline) + 0·85 (≥ T3) + 1·10 (poor differentiation) + 1·20 (stable disease on CT) + 1·00 (tumour volume > 14 cm^3^) + 0·51 (invasion on CT).

When the five variables were positive, the likelihood of CRM positivity was 82 per cent.

Of the 155 patients included in the main cohort, 35 (22·6 per cent) developed locoregional recurrence. Median time to locoregional recurrence was 16·5 months with a median follow‐up of 25·3 months. Of these, 19 (54 per cent) occurred in association with synchronous systemic recurrence. There was a higher rate of locoregional recurrence in patients with a positive CRM although it was not statistically significant (29 per cent *versus* 18 per cent for CRM negativity; *P* = 0·092) (*Table* 1). There were higher rates of locoregional recurrence for pT3–4 category (26 per cent *versus* 18 per cent for pT1–2), poorly differentiated tumours (25 per cent *versus* 20 per cent for moderately/well differentiated tumours), invasion on CT (31 per cent *versus* 18 per cent for no invasion) and tumours with a volume of at least 14 cm^3^ (24 per cent *versus* 21 per cent for those smaller than 14 cm^3^), although these differences were not statistically significant. Variables associated with significantly higher rates of locoregional recurrence were pN status (pN0 11, 27 and 32 per cent for pN0, pN1 and pN2–3 respectively; *P* = 0·020), lymphovascular invasion (30 per cent *versus* 12 per cent for no invasion; *P* = 0·007) and poor response to chemotherapy (Mandard score 4–5 29 per cent *versus* 10 per cent for score 1–3; *P* = 0·006).

## Discussion

This study has identified that clinical tumour status (cT3 and above) and grade (poor differentiation) independently predict a positive CRM in patients with oesophageal adenocarcinoma before chemotherapy. After neoadjuvant chemotherapy, the addition of CT‐assessed lack of response (stable or progressive disease), postchemotherapy tumour volume on CT (at least 14 cm^3^) and invasion of adjacent structures on CT increased the accuracy of a threatened CRM prediction model with an AUC of 0·76. Patients with all five parameters had an 82 per cent chance of CRM positivity.

A positive CRM is a major determinant of outcome following surgical resection^8^, ^9^, ^10^. However, prediction of margin involvement before oesophagectomy has inherent challenges. There is no specific anatomical dissection plane that is easily visualized on preoperative imaging, in contrast to the mesorectal fascia in rectal cancer. There is also no serosal layer on the oesophagus, which poses a challenge when determining tumour resectability before surgical exploration. Imaging after chemotherapy or chemoradiotherapy cannot reliably differentiate viable tumour from fibrosis or inflammation.

There are some methodological limitations to this study. The use of non‐randomized data from a single institution must be interpreted with caution. Both TTO and THO procedures were performed. Although there could be potential bias due to the variation in surgical techniques, selection of patients for each approach mandated dissection of the primary tumour under direct vision. A previous study^22^ at the authors' institution did not find an overall difference in terms of survival, recurrence rates or margin positivity between these two techniques. Although CRM rates appeared high, the exclusion of patients with early tumours from this selected cohort, use of chemotherapy rather than chemoradiotherapy, and adoption of the Royal College of Pathologists' definition of a positive margin may all have contributed.

Patients in this cohort were staged clinically using a combination of EUS, PET and CT. After chemotherapy, CT and EUS are often inaccurate in reassessing tumour status^23^. The radiological variables used in this study were CT‐based, as this is the most commonly used imaging modality despite certain limitations in accuracy. The improved performance of the postsurgical model using pathological data confirms that that the accuracy of preoperative CRM prediction may increase with further improvements in clinical staging. With the introduction of clinical PET scanners integrated with MRI, which has higher inherent soft‐tissue contrast than CT, the accuracy of preoperative CRM prediction may increase in the future^23^. The derived models still require external validation.

The sensitivity of the prediction models was lowest at diagnosis, when decisions are traditionally made regarding neoadjuvant treatment. This sensitivity improved significantly after chemotherapy, and therefore has the potential to select patients with a higher likelihood of a positive CRM for treatment intensification with radiotherapy before resection. RCT evidence would be needed to determine whether this escalation improved survival in patients at risk of CRM positivity following neoadjuvant chemotherapy.

A previous study^20^ from the authors' institution showed that a positive CRM did not independently increase the risk of locoregional recurrence. This was in contrast to the findings of a large study that showed increased rates of locoregional recurrence in patients with an R1 resection margin, albeit using the College of American Pathologists' definition of a positive CRM, defined as tumour present at the cut margin^9^. Ultimately, CRM‐positive patients are still more likely to die from systemic disease than from an isolated local recurrence, a pattern seen in patients treated by either chemotherapy or chemoradiotherapy^14^, ^24^. This emphasizes the importance of effective systemic therapy in the treatment of oesophageal cancer.

The preoperative model encompassed five factors that would logically imply a threatened margin, namely: depth of tumour invasion (T3–4), aggressive biology (poor differentiation), how well the tumour has responded to treatment (CT estimation of tumour response), tumour size (tumour volume) and evidence of local invasion by involvement of adjacent structures (CT invasion). The fact that these same factors also independently influence overall survival^25^, ^26^, ^27^, ^28^, ^29^ suggests that a positive resection margin is a surrogate for aggressive tumour biology and not simply a measure of inadequate locoregional clearance. This conclusion has also been reached by others^9^, ^30^.

The idea of selecting at‐risk patients for more tailored therapeutic strategies seems logical. The use of imaging to predict CRM involvement has been effective in tailoring treatments and improving CRM and local recurrence rates in rectal cancer^19^, ^20^. The use of radiotherapy to intensify treatment is less well explored in oesophageal cancer. The MUNICON II study^31^ assessed such a strategy for patients who did not respond to chemotherapy on PET, but showed no additional survival benefit for radiotherapy, largely because of high rates of systemic relapse. The present study indicates that, following neoadjuvant chemotherapy, a predictive model based on cT status, tumour volume, poor differentiation, lack of response following chemotherapy, and radiological invasion could identify patients at high risk of CRM positivity. RCT evidence would be needed to determine whether the addition of radiotherapy in the neoadjuvant setting might influence survival in these patients.

## Collaborators

The following members of the Guy's and St Thomas' Oesophago‐Gastric Research Group are co‐authors of this study: S. Ngan, A. Qureshi (Department of Oncology Guy's and St Thomas' Hospital, London, UK); H. Deere, M. Green, F. Chang, U. Mahadeva, B. Gill‐Barman, S. George (Department of Cellular Pathology, Guy's and St Thomas' Hospital, London, UK); J. Dunn, S. Zeki, J. Meenan (Department of Gastroenterology, Guy's and St Thomas' Hospital, London, UK); O. Hynes, G. Tham, C. Iezzi (Department of Surgery, Guy's and St Thomas' Oesophago‐Gastric Centre, London, UK).

## Supporting information


**Table S1** Univariable and multivariable analysis of the association between radiological parameters and the risk of positive circumferential resection margin involvement
