# Supplementary material for: Prediction of a positive circumferential resection margin at surgery following neoadjuvant chemotherapy for adenocarcinoma of the oesophagus
Source: BJS Open. 2019 Aug 22;3(6):767–76. doi: 10.1002/bjs5.50211 (PMC6887675; doi:10.1002/bjs5.50211)
Supplement: Supplementary file 1 — Table S1 Univariable and multivariable analysis of the association between radiological parameters and the risk of positive circumferential resection margin involvement [file BJS5-3-767-s001.doc]

**BJS5_50211**

# **Prediction of a positive circumferential resection margin at surgery following neoadjuvant chemotherapy for adenocarcinoma of the oesophagus**

**W. R. C. Knight, C. Yip, A. Jacques, N. Griffin, J. Zylstra, W. Wulaningsih, M. Van Hemelrijck, N. Maisey, A. Gaya, C. R. Baker, M. Kelly, J. A. Gossage, J. Largergren, D. Landau, V. Goh, A. R. Davies**

**Table S1** Univariable and multivariable analysis of the association between radiological parameters and the risk of positive circumferential resection margin involvement

|  |  | | | ***Univariable*** | | | ***Multivariable*** | | |
| --- | --- | --- | --- | --- | --- | --- | --- | --- | --- |
| **Parameters** | **Number of patients** | | | **OR** | **95% CI** | ***p-*value** | **OR** | **95% CI** | ***p-*value** |
|  | **Total** | **Negative CRM** | **Positive CRM** |  |  |  |  |  |  |
| **Age (median)** |  |  |  |  |  |  |  |  |  |
| < 61.5 | 34 | 18 | 16 |  |  |  |  |  |  |
| ≥ 61.5 | 34 | 23 | 11 | 0.5 | (0.2 - 1.6) | 0.320 |  |  |  |
| **Sex** |  |  |  |  |  |  |  |  |  |
| Female | 10 | 7 | 3 |  |  |  |  |  |  |
| Male | 58 | 34 | 24 | 1.6 | (0.3 - 10.8) | 0.730 |  |  |  |
| **Neoadjuvant chemotherapy** |  |  |  |  |  |  |  |  |  |
| No | 15 | 11 | 4 |  |  |  |  |  |  |
| Yes | 53 | 30 | 23 | 2.1 | (0.5 - 10.2) | 0.370 |  |  |  |
| **Invasion of adjacent structures** |  |  |  |  |  |  |  |  |  |
| No | 55 | 37 | 18 |  |  |  |  |  |  |
| Yes | 13 | 4 | 9 | 4.5 | (1.1 - 22.9) | **0.030** |  |  |  |
| **Radiologist’s prediction of CRM status** |  |  |  |  |  |  |  |  |  |
| Negative | 43 | 30 | 13 |  |  |  |  |  |  |
| Positive | 25 | 11 | 14 | 2.9 | (0.9 - 9.3) | **0.040** |  |  |  |
| **Primary tumour visibility on CT** |  |  |  |  |  |  |  |  |  |
| Visible | 58 | 33 | 25 |  |  |  |  |  |  |
| Not visible | 10 | 8 | 2 | 0.3 | (0.0 - 1.9) | 0.290 |  |  |  |
| **Presence of mediastinal fat** |  |  |  |  |  |  |  |  |  |
| Yes | 57 | 33 | 24 |  |  |  |  |  |  |
| No | 11 | 8 | 3 | 0.5 | (0.1 - 2.5) | 0.510 |  |  |  |
| **Margin well defined** |  |  |  |  |  |  |  |  |  |
| No | 39 | 23 | 16 |  |  |  |  |  |  |
| Yes | 29 | 18 | 11 | 0.9 | (0.3 - 2.6) | 1.000 |  |  |  |
| **Circumferential fat plane present around tumour** |  |  |  |  |  |  |  |  |  |
| No | 40 | 21 | 19 |  |  |  |  |  |  |
| Yes | 28 | 20 | 8 | 0.4 | (0.1 - 1.4) | 0.140 |  |  |  |
| **Contact with adjacent structures** |  |  |  |  |  |  |  |  |  |
| No | 28 | 21 | 7 |  |  |  |  |  |  |
| Yes | 40 | 20 | 20 | 3 | (0.9 - 10.2) | **0.050** |  |  |  |
| **Circumferential aortic contact >90°^a^** |  |  |  |  |  |  |  |  |  |
| No | 60 | 39 | 21 |  |  |  |  |  |  |
| Yes | 8 | 2 | 6 | 5.4 | (0.9 - 59.6) | **0.050** |  |  |  |
| **Diaphragmatic contact** |  |  |  |  |  |  |  |  |  |
| No | 67 | 40 | 27 |  |  |  |  |  |  |
| Yes | 1 | 1 | 0 | 0 | (0.0 - 59.2) | 1.000 |  |  |  |
| **Pleural thickening** |  |  |  |  |  |  |  |  |  |
| No | 62 | 40 | 22 |  |  |  |  |  |  |
| Yes | 6 | 1 | 5 | 8.8 | (0.9 - 49.3) | **0.030** |  |  |  |
| **Maximum wall thickness** **(median, cm)** |  |  |  |  |  |  |  |  |  |
| ≤ 1.65 | 34 | 24 | 10 |  |  |  |  |  |  |
| > 1.65 | 27 | 15 | 12 | 1.9 | (0.6 - 6.3) | 0.290 |  |  |  |
| **Presence of enlarged lymph nodes** |  |  |  |  |  |  |  |  |  |
| No | 45 | 31 | 14 |  |  |  |  |  |  |
| Yes | 23 | 10 | 13 | 2.8 | (0.9 - 9.2) | 0.070 | 2.4 | 0.8 - 7.7 | 0.130 |
| **Tumour length (median, cm)** |  |  |  |  |  |  |  |  |  |
| ≤ 5.5 | 29 | 16 | 13 |  |  |  |  |  |  |
| > 5.5 | 26 | 15 | 11 | 0.9 | (0.3 - 3.0) | 1.000 |  |  |  |
| **Largest transaxial primary tumour dimension** (**LD) (median, cm)** |  |  |  |  |  |  |  |  |  |
| ≤ 2.6 | 38 | 29 | 9 |  |  |  |  |  |  |
| > 2.6 | 30 | 12 | 18 | 4.7 | (1.5 - 15.8) | **0.003** | 2.9 | 0.9 - 9.1 | 0.070 |
| **CT invasion** (including invasion of adjacent structures, pleura thickening and circumferential aortic  contact >90° |  |  |  |  |  |  |  |  |  |
| No | 50 | 36 | 14 |  |  |  |  |  |  |
| Yes | 18 | 5 | 13 | 6.7 | 2.0-22.2 | 0.002 | 4.4 | (1.2-16.1) | 0.003 |
